# Association between heavy metals exposure and infertility among American women aged 20–44 years: A cross-sectional analysis from 2013 to 2018 NHANES data

**DOI:** 10.3389/fpubh.2023.1122183

**Published:** 2023-02-14

**Authors:** Jie Lin, Xiaoyan Lin, Jiahui Qiu, Xiumi You, Jinbang Xu

**Affiliations:** ^1^Department of Traditional Chinese Medicine, Fujian Maternity and Child Health Hospital, College of Clinical Medicine for Obstetrics & Gynecology and Pediatrics, Fujian Medical University, Fuzhou, Fujian, China; ^2^College of Economics and Management, Fujian Agriculture and Forestry University, Fuzhou, Fujian, China

**Keywords:** infertility, arsenic, lead, cadmium, NHANES

## Abstract

**Background:**

Infertility has been confirmed as a significant medical and social problem. Heavy metal exposure refers to a risk factor for infertility, which is capable of damaging the reproductive system of males and females. However, heavy metal exposure and female infertility have rarely been investigated. The aim of this study was to analyze the association between heavy metal exposure and female infertility.

**Methods:**

A cross-sectional study using data from three cycles of the National Health and Nutrition Examination Survey (NHANES, 2013–2018) was performed. Female infertility was evaluated by positive responses to question rhq074 in the questionnaire. Cadmium (Cd), lead (Pb), mercury (Hg), and arsenic (As) levels in blood or urine were examined by inductively coupled plasma mass spectrometry. The correlation between heavy metal and female infertility was analyzed through weighted logistic regression.

**Results:**

A total of 838 American women aged 20–44 years were covered in the study. Among all participants, 112 (13.37%) women were subjected to infertility. Urinary Cd and urinary As levels were significantly higher in infertile women than in control women (*P* < 0.05, *P* < 0.05). Urinary As showed a positive correlation with the prevalence of female infertility, and the risk of infertility rose with the increase of urinary As levels (*P* for trend = 0.045). Urinary Cd was associated with female infertility in some weighted logistic regression (Crude, Q2: OR = 3.99, 95% CI 1.82, 8.74, Q3: OR = 2.90, 95% CI 1.42, 5.92. Model 1, Q2: OR = 3.68, 95% CI 1.64, 8.27, Q3: OR = 2.33, 95% CI 1.13, 4.48. Model 2, Q2: OR = 4.11, 95% CI 1.63, 10.07, Q3: OR = 2.44, 95% CI 1.07, 5.53. Model 3, Q2: OR = 3.77, 95% CI 1.52, 9.35). Moreover, blood Pb (OR = 1.52, 95% CI 1.07, 2.16), urinary Pb (OR = 1.68, 95% CI 1.11, 2.55), and urinary As (OR = 1.02, 95% CI 1.00, 1.03) were positively correlated with the risk of infertility in women aged 35–44 years. The blood Pb (OR = 1.67, 95% CI 1.16, 2.40, 2.49) and urinary Pb (OR = 1.54, 95% CI 1.00, 2.38) in women with BMI ≥25 were positively correlated with the risk of infertility.

**Conclusions:**

Urinary As was significantly associated with female infertility, and the risk of infertility increased with higher urinary As levels. To some extent, urinary Cd was correlated with infertility. Blood/urine Pb was related to infertility in advanced age and overweight/obese women. The results of this study need to be further validated in future prospective studies.

## Introduction

Infertility is defined as the failure of a man and a woman to conceive after living together for over 12 months, having regular sexual intercourse and neither of them using contraception, as indicated by the latest regulations of the World Health Organization (1). Infertility refers to a gynecological condition, a group of states of fertility disorders caused by multiple causes and an adverse reproductive health event for women of childbearing age. It is estimated that infertility affects up to 186 million people worldwide (2). Nearly 6–15% of couples of childbearing ages around the world suffer from infertility (2). In some developing countries, the prevalence of infertility reaches up to 30% (3, 4). Infertility has been confirmed as a specific reproductive health defect that causes individuals mental disorders (e.g., insomnia, anxiety, depression, eating disorders, and addictions) (5) while triggering divorce. The divorce rate of infertile couples is 2.2 times higher than the normal population, as suggested by a survey from China (6). As a result, infertility has become a significant medical and social problem. The leading causes of infertility comprise ovulatory dysfunction, male factor infertility, and tubal disease. Nevertheless, poor lifestyles and environmental factors can impair fertility (7). To be specific, adverse environmental factors (e.g., heavy metal exposure) can impair the reproductive system in both men and women (8, 9).

Heavy metals are metals with a density >5 g/cm^3^ [e.g., gold, silver, mercury (Hg), copper, lead (Pb), cadmium (Cd), and chromium]. Heavy metal pollution refers to Hg, Pb, Cd, chromium, and metal-like arsenic (As), which are non-essential and highly toxic to humans (10). Acute poisoning by heavy metals is rare. The general population's whole-blood concentrations of Pb and Hg are < 20 and 25 μg/L, respectively (11). When the blood As level exceeds 100 μg/L, it produces toxic effects on tissues such as the gastrointestinal tract, nerves, kidneys, etc (11). The above heavy metals are often found in the atmosphere, food, drinking water, and cosmetics. Excessive exposure to Cd, Pb, As, and Hg in the everyday environment may be toxic to human reproduction and development (9, 12–14). The main toxic effects of Cd, Pb, Hg, and As on the reproductive endocrine system comprise the promotion of oxidative stress, reduction of follicle-stimulating hormone, reduction of luteinizing hormone, reduction of follicular growth, promotion of follicular atresia and disruption of the estrous cycle (15–20).

Environmental and occupational exposure to metals impairs female reproductive health by affecting the reproductive system at all strata of regulation and functions, resulting in female infertility, menstrual disorders, spontaneous abortion, endometriosis, endometrial cancer, breast cancer, etc (21). A previously cross-sectional study suggested that blood Cd and Pb were positively correlated with infertility in US women (22). Another study reported that blood Pb and As concentrations in infertile women in Taiwan, China was significantly higher than those in pregnant (14). Pregnancy was associated with significantly reduced Cd, Pb, and Hg (23). Basic and clinical studies have been reported on the adverse effects of Cd, Pb, Hg, and As exposure on female infertility. Although many studies have been conducted in animals, rare observational studies have been conducted in humans. Heavy metals can accumulate in human blood, urine, hair, follicles, embryos, testes, liver, kidneys, and other tissues, thus exerting adverse effects. Of these, blood and urine are non-invasive and readily available clinical samples. In this study, we aimed to evaluate whether Cd, Pb, Hg, and As are correlated with self-reported infertility in American women by comparing their blood and urine levels.

## Methods

### Data source and study population

All data originated from the National Health and Nutrition Examination Survey (NHANES) database. NHANES refers to a population-based cross-sectional survey that collects information regarding the health and nutrition of the U.S. household population. The survey was planned to review a nationally representative sample of ~5,000 individuals per year in a 2-year survey cycle. The survey results were adopted to determine the prevalence and risk factors for relevant diseases. All protocols implemented by NHANES were reviewed and approved by the National Centers for Health Statics Institutional Review Board (Continuation of Protocol #2011-17 and Protocol #2018-01). All participants in the study data collection signed an informed consent form.

**Figure 1:**
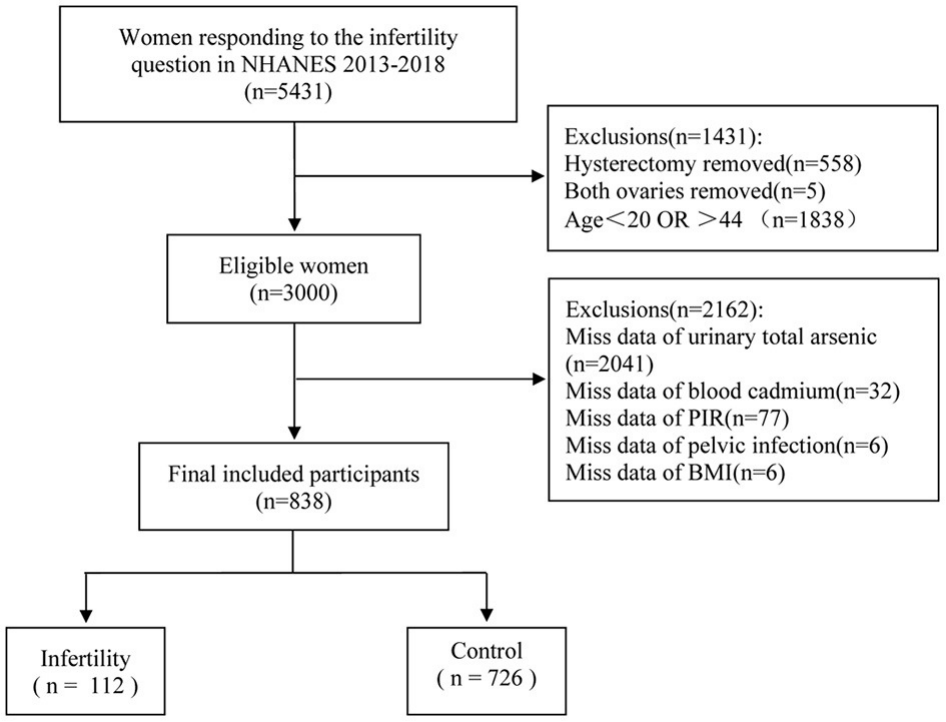
Flow chart for participants recruitment of this study, NHANES 2013–2018. PIR, ratio of family income to poverty; BMI, body mass index.

The NHANES data from three survey cycles in 2013–2014, 2015–2016, and 2017–2018 were selected for this cross-sectional study. First, women (*n* = 3,707) answering the infertility question (rhq074) were covered in the analysis. However, women with a history of hysterectomy (*n* = 558), bilateral oophorectomy (*n* = 5), and age >20 or < 44 years (*n* = 1838) were excluded, as well as women without information regarding urinary AS, blood Cd, the ratio of family income to poverty (PIR), pelvic infection, and body mass index (BMI) (*n* = 2156). A final sample of 838 women was included. Figure 1 illustrates the sample selection process.

### Dependent variable

Infertility was determined by a woman's response to the question, “have you ever attempted to become pregnant over at least a year, without becoming pregnant?” in the reproductive health questionnaire section of rhq074. Women answering “yes” were considered infertility, whereas women answering “no” were considered normal.

### Independent variable

Heavy metal concentration data were derived from the laboratory section of the NHANES database. Whole blood and urine samples were collected by physicians at the NHANES mobile examination center, processed, frozen at −30°C, and then shipped to the National Center for Environmental Health, Centers for Disease Control and Prevention, Atlanta, GA, for analysis. The concentrations of blood Cd, blood Pb, blood Hg, urinary Cd, urinary Pb, and urinary As were examined through inductively coupled plasma mass spectrometry. The reported results of all assays conformed to the Laboratory Sciences Division's quality control and quality assurance performance standards for accuracy and precision, which were similar to the Westgard rule (24–26). The lower limits of detection for Cd, Pb, and Hg in blood reached 0.10, 0.07, and 0.28 μg/dL, respectively. The lower limits of detection for Cd, Pb, and As in urine were obtained as 0.036, 0.03, and 0.23 μg/L, respectively. All analytical results achieved using NHANES data processing methods were equal to or exceeded the detection limits.

### Covariates

In this study, covariates comprised age, ethnicity, education, BMI, marital status, regular menstrual periods, PIR, smoking history, as well as pelvic infection. The inclusion of the above covariates was determined professionally and after reading the existing research (7, 27, 28). These covariates were derived from the demographic, the examination, the reproductive health questionnaire, and the smoking questionnaire section of the NHANES database.

### Statistical analysis

Data originated from the NHANES for three cycles (2013–2014, 2015–2016, and 2017–2018). A descriptive analysis was conducted on the demographic and measurement indicators of the study population. These indicators were assigned to two groups in accordance with whether they were subjected to infertility or not. Continuous variables are expressed as mean ± standard error (Mean ± S.E.), and a *t*-test was performed for comparison between groups. Categorical variables are expressed as frequency (composition ratio) [*n* (%)], and the chi-square test was performed for comparison between groups.

Weighted multivariate logistic regression was conducted to evaluate the correlation between heavy metal levels and female infertility. Blood Cd, blood Pb, blood Hg, urinary Cd, urinary Pb, and urinary As concentrations were classified according to quartiles (Q1, Q2, Q3, and Q4). The correlation between heavy metals and the risk of infertility was analyzed through univariate and multifactorial logistic regression analyses, with the Q1 category as a reference, and the results are expressed as odds ratio (OR) and 95% confidence interval (95% CI). A single-factor logistic regression analysis was first conducted in crude model, followed by a multi-factor logistic regression analysis for model 1, model 2, and model 3. To be specific, Model 1 was adjusted for age. Model 2 was adjusted for ethnicity, education, marital status, and PIR on top of model 1. Moreover, model 3 was adjusted for BMI, regular menstrual periods, pelvic infection, and smoking history on top of model 2. Furthermore, subgroup analysis was conducted based on age and BMI.

Data extraction, screening, and statistical analysis were conducted using R 4.1.2 (Institute of Statistics and Mathematics, Vienna, Austria). α = 0.05 served as the criterion for significance.

## Results

### Demographic characteristics of participants

In this study, a total of 838 women were covered, which comprised 112 infertile women and 726 control women. Table 1 lists the basic characteristics of the study population. Age and BMI were significantly higher in infertile women than those in control women (*P* < 0.05, *P* < 0.05). Marital status was significantly different between infertile women and control women (*P* = 0.002). There was no significant difference in ethnicity, education, PIR, regular menstrual periods, pelvic infection, and smoking history (all *P* > 0.05).

**Table 1:** Characteristics of the study population.

**Variables**	**Total (*n* = 838)**	**Infertility (*n* = 112)**	**Control (*n* = 726)**	***P*-value**
Age, years, mean (S.E.)	31.60 (0.23)	34.36 (0.80)	31.27 (0.23)	< 0.001^*^
Ethnicity, *n* (%)				0.14
Mexican American	161 (19.21)	29 (19.38)	132 (12.25)	
Non-hispanic white	276 (32.94)	31 (48.12)	245 (56.36)	
Non-hispanic black	167 (19.93)	28 (18.98)	139 (13.25)	
Other Hispanic	84 (10.02)	7 (4.74)	77 (7.40)	
Other race	150 (17.90)	17 (8.78)	133 (10.74)	
Education, *n* (%)				0.51
< 9th grade	39 (4.65)	5 (3.94)	34 (2.86)	
9–11th grade	78 (9.31)	12 (9.01)	66 (8.06)	
High school graduate/GED or equivalent	170 (20.29)	17 (14.15)	153 (21.59)	
Some college or AA degree	324 (38.66)	48 (38.49)	276 (36.81)	
College graduate or above	227 (27.09)	30 (34.42)	197 (30.68)	
Marital status, *n* (%)				0.002^*^
Married	364 (43.44)	65 (62.50)	299 (41.46)	
Widowed	3 (0.36)	1 (0.68)	2 (0.10)	
Divorced	47 (5.61)	4 (3.08)	43 (5.82)	
Separated	28 (3.34)	4 (3.16)	24 (2.70)	
Never married	269 (32.1)	22 (19.29)	247 (34.05)	
Living with partner	127 (15.16)	16 (11.30)	111 (15.88)	
PIR, mean (S.E.)	2.62 (0.08)	2.70 (0.23)	2.61 (0.09)	0.70
BMI, kg/m^2^, Mean (S.E.)	29.60 (0.43)	31.52 (0.88)	29.34 (0.45)	0.02^*^
Regular menstrual periods, *n* (%)				0.07
Yes	773 (92.24)	101 (85.32)	672 (92.58)	
No	65 (7.76)	11 (14.68)	54 (7.42)	
Pelvic infection, *n* (%)				0.12
Yes	44 (5.25)	11 (7.86)	33 (3.71)	
No	794 (94.75)	101 (92.14)	693 (96.29)	
Smoking history, *n* (%)				0.52
Yes	259 (30.91)	38 (39.34)	221 (34.87)	
No	579 (69.09)	74 (60.66)	505 (65.13)	

^*^P < 0.05. S.E., standard error; GED, general educational development; AA, associates; PIR, ratio of family income to poverty; BMI, body mass index.

### Cd, Pb, Hg, and As differences between infertile and control women

As depicted in Table 2, urinary Cd and urinary As levels were significantly higher in infertile women than in control women (*P* < 0.05, *P* < 0.05). However, there were no measurable differences in blood Cd, blood Pb, blood Hg and urinary Pb levels between infertile women and control women (all *P* > 0.05).

**Table 2:** Cd, Pb, Hg, and As differences between infertile and control women.

**Heavy metals**	**Total (*n* = 838)**	**Infertility (*n* = 112)**	**Control (*n* = 726)**	***P*-value**
**Heavy metals in blood, mean (S.E.)**				
Cd, μg/L	0.45 (0.03)	0.49 (0.07)	0.45 (0.03)	0.61
Pb, μg/dL	0.70 (0.04)	0.89 (0.19)	0.67 (0.03)	0.27
Hg, μg/L	1.15 (0.07)	1.08 (0.14)	1.15 (0.07)	0.65
**Heavy metals in urine, mean (S.E.)**				
Cd, μg/L	0.20 (0.01)	0.25 (0.02)	0.20 (0.01)	0.03^*^
Pb, μg/L	0.34 (0.03)	0.48 (0.16)	0.32 (0.02)	0.30
As, μg/L	12.99 (1.38)	21.19 (4.22)	11.89 (1.42)	0.04^*^

^*^P < 0.05. S.E., standard error; Cd, cadmium; Pb, lead; Hg, mercury; As, arsenic.

### Correlation between Cd, Pb, Hg, As and female infertility

Logistic regression results for Cd, Pb, Hg, As and infertile females are listed in Table 3. In this analysis, the participants were grouped in accordance with interquartile range (IQR) of heavy metal concentration (Q1: 0–25%, Q2: >25–50%, Q3: >50–75%, Q4: >75–100%), and the Q1 group served as a reference group for the analysis. The univariate analysis illustrated that urinary Cd levels were correlated with infertility in the Q3 group (OR = 3.99, 95% CI 1.82, 8.74) and Q4 group (OR = 2.90, 95% CI 1.42, 5.92), *P* for trend = 0.001. Urinary As levels were associated with infertility in the Q4 group (OR = 2.58, 95% CI 1.45, 4.59), *P* for trend = 0.01. Blood Cd levels were correlated with infertility in the Q2 group (OR = 2.12, 95% CI 1.03, 4.38), *P* for trend = 0.59. Blood Pb levels were related with infertility in the Q3 group (OR = 2.86, 95% CI 1.37, 5.93), *P* for trend = 0.34. Urinary Pb levels were correlated with infertility in the Q3 group (OR = 2.13, 95% CI 1.02, 4.46), *P* for trend = 0.09. However, blood Hg was not correlated with infertility.

**Table 3:** Odds ratios (95% confidence intervals) of infertility across quartiles of Cd, Pb, Hg, and As, NHANES 2013–2018.

	**Crude**	**Model 1**	**Model 2**	**Model 3**
**Blood Cd**, μ**g/L**				
Q1 (0.07, 0.18)	Ref.	Ref.	Ref.	Ref.
Q2 (0.18, 0.28)	2.12 (1.03, 4.38)^*^	1.90 (0.91, 3.97)	1.77 (0.82, 3.83)	1.69 (0.78, 3.67)
Q3 (0.28, 0.488)	1.52 (0.68, 3.39)	1.27 (0.53, 3.01)	1.12 (0.48, 2.89)	1.17 (0.46, 2.93)
Q4 (0.488, 5.14)	1.6 (0.68, 3.80)	1.38 (0.56, 3.40)	1.56 (0.60, 4.07)	1.349 (0.49, 3.71)
*P* for trend	0.59	0.85	0.55	0.83
**Blood Pb**, μ**g/dL**				
Q1 (0.07, 0.39)	Ref.	Ref.	Ref.	Ref.
Q2 (0.39, 0.55)	1.98 (0.95, 4.12)	1.94 (0.91, 4.14)	2.02 (0.92, 4.42)	2.08 (0.94, 4.62)
Q3 (0.55, 0.808)	2.86 (1.37, 5.93)^*^	2.55 (1.18, 5.49)^*^	2.67 (1.20, 5.93)^*^	2.62 (1.19, 5.77)^*^
Q4 (0.808, 7.36)	1.70 (0.73, 3.93)	1.38 (0.61, 3.14)	1.53 (0.67, 3.51)	1.53 (0.66, 3.56)
*P* for trend	0.34	0.76	0.57	0.59
**Blood Hg**, μ**g/L**				
Q1 (0.2, 0.35)	Ref.	Ref.	Ref.	Ref.
Q2 (0.35, 0.665)	1.79 (0.89, 3.64)	1.71 (0.83, 3.49)	1.66 (0.78, 3.53)	1.60 (0.73, 3.49)
Q3 (0.665, 1.34)	1.76 (0.81, 3.86)	1.52 (0.67, 3.46)	1.54 (0.62, 3.81)	1.58 (0.62, 4.00)
Q4 (1.34, 20.39)	1.48 (0.70, 3.17)	1.32 (0.63, 2.75)	1.27 (0.55, 2.92)	1.30 (0.55, 3.07)
*P* for trend	0.60	0.86	0.95	0.89
**Urinary Cd**, μ**g/L**				
Q1 (0.025, 0.065)	Ref.	Ref.	Ref.	Ref.
Q2 (0.065, 0.141)	1.41 (0.66, 3.02)	1.38 (0.64, 2.98)	1.50 (0.65, 3.45)	1.45 (0.65, 3.27)
Q3 (0.141, 0.28)	3.99 (1.82, 8.74)^*^	3.68 (1.64, 8.27)^*^	4.11 (1.63, 10.37)^*^	3.77 (1.52, 9.35)^*^
Q4 (0.28, 4.47)	2.90 (1.42, 5.92)^*^	2.33 (1.13, 4.83)^*^	2.44 (1.07, 5.53)^*^	2.06 (0.91, 4.66)
*P* for trend	0.001^*^	0.02^*^	0.03^*^	0.09
**Urinary Pb**, μ**g/L**				
Q1 (0.02, 0.12)	Ref.	Ref.	Ref.	Ref.
Q2 (0.12, 0.23)	1.59 (0.80, 3.14)	1.66 (0.85, 3.26)	1.67 (0.81, 3.42)	1.64 (0.81, 3.30)
Q3 (0.23, 0.4)	2.13 (1.02, 4.46)^*^	2.16 (1.02, 5.55)^*^	2.20 (0.96, 5.06)	1.96 (0.86, 4.50)
Q4 (0.4, 8.25)	1.76 (0.94, 3.29)	1.76 (0.94, 3.29)	1.75 (0.86, 3.57)	1.65 (0.76, 3.61)
*P* for trend	0.09	0.10	0.13	0.24
**Urinary As**, μ**g/L**				
Q1 (0.18, 2.993)	Ref.	Ref.	Ref.	Ref.
Q2 (2.993, 5.96)	1.29 (0.69, 2.42)	1.50 (0.79, 2.87)	1.56 (0.78, 3.13)	1.41 (0.69, 2.89)
Q3 (5.96, 11.678)	1.66 (0.86, 3.21)	1.85 (0.95, 3.60)	1.84 (0.86, 3.90)	1.73 (0.81, 3.68)
Q4 (11.678, 458.56)	2.58 (1.45, 4.59)^*^	2.73 (1.51, 4.96)^*^	2.81 (1.35, 5.83)^*^	2.76 (1.33, 5.70)^*^
*P* for trend	0.01^*^	0.01^*^	0.049^*^	0.045^*^

^*^P < 0.05. Crude, no covariate was adjusted. Model 1 adjusted for age. Model 2 adjusted for age, ethnicity, education, marital status, and PIR. Model 3 adjusted for age, ethnicity, race, education, marital status, PIR, BMI, regular menstrual periods, pelvic infection, and smoking history. Cd, cadmium; Pb, lead; Hg, mercury; As, arsenic.

After adjustment for age in Model 1, the relationship between urinary Cd, urinary As and infertility were still robust (*P* for trend = 0.02, *P* for trend = 0.01). Blood Pb (OR = 2.55, 95% CI 1.18, 5.49) and urinary Pb (OR = 2.16, 95% CI 1.02, 5.55) levels were still associated with infertility in the Q3 group. However, no correlation was found between blood Cd, blood Hg and infertility.

After adjustment for age, ethnicity, education, marital status, and PIR in Model 2, urinary Cd and urinary As were positively correlated with female infertility (*P* for trend = 0.03, *P* for trend = 0.049). Blood Pb levels were still associated with infertility in the Q3 group (OR = 2.67, 95% CI 1.20, 5.93). However, blood Cd, blood Hg and urinary Pb were not markedly related with infertility.

After adjustment for age, ethnicity, education, marital status, PIR, BMI, regular menstrual periods, smoking history and pelvic infection in Model 3, urinary As was positively associated with female infertility (*P* for trend = 0.045). Blood Pb (OR = 2.62, 95% CI 1.19, 5.77) and urinary Cd (OR = 3.77, 95% CI 1.52, 9.35) levels were correlated with infertility in the Q3 group. However, blood Cd, blood Hg and urinary Pb were still unrelated to infertility.

### Stratification analysis

**Figure 2:**
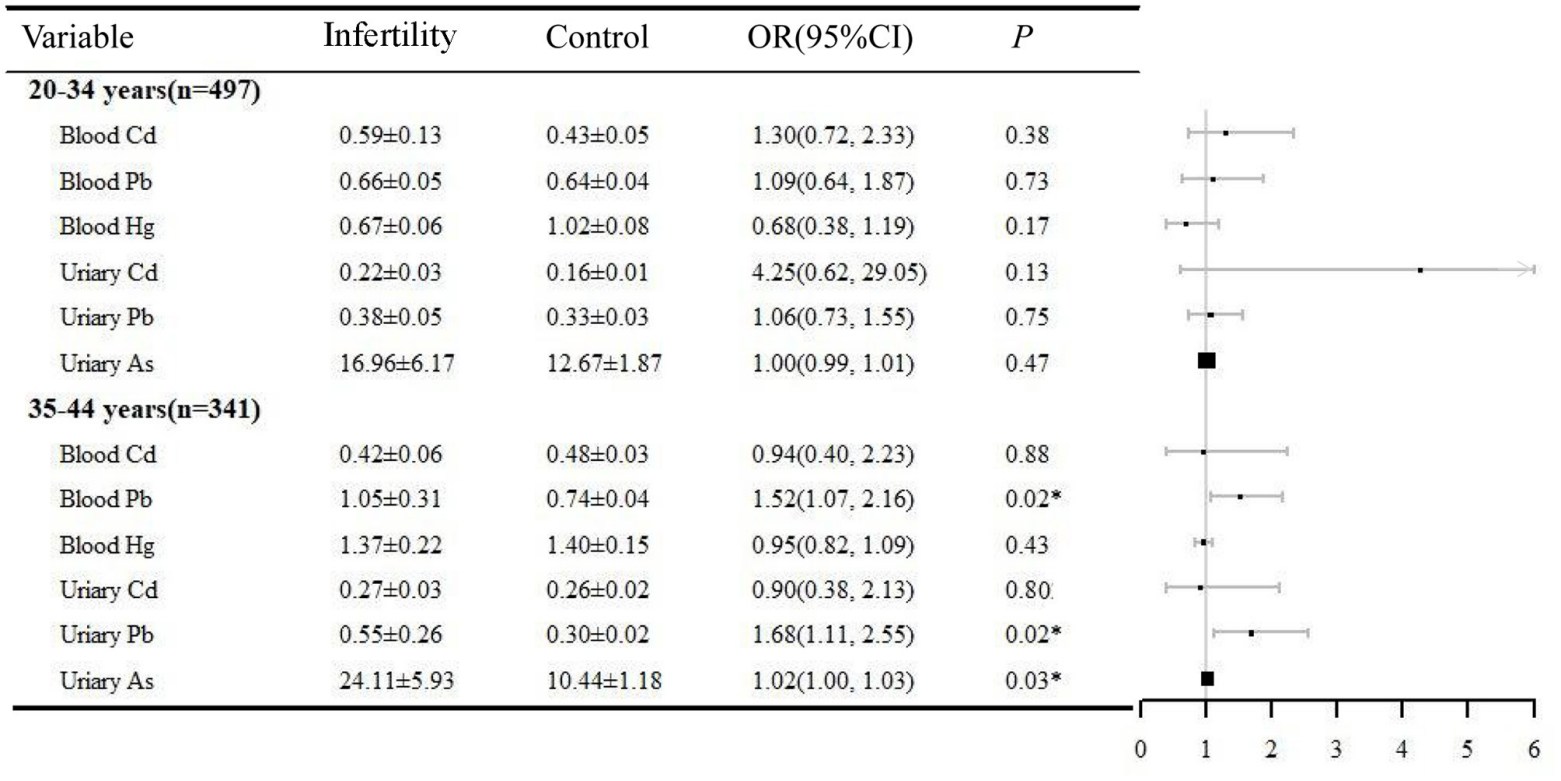
Age-stratified analysis of the correlation between heavy metals and infertility, NHANES 2013–2018. *P < 0.05. OR, odds ratio; CI, confidence interval.

**Figure 3:**
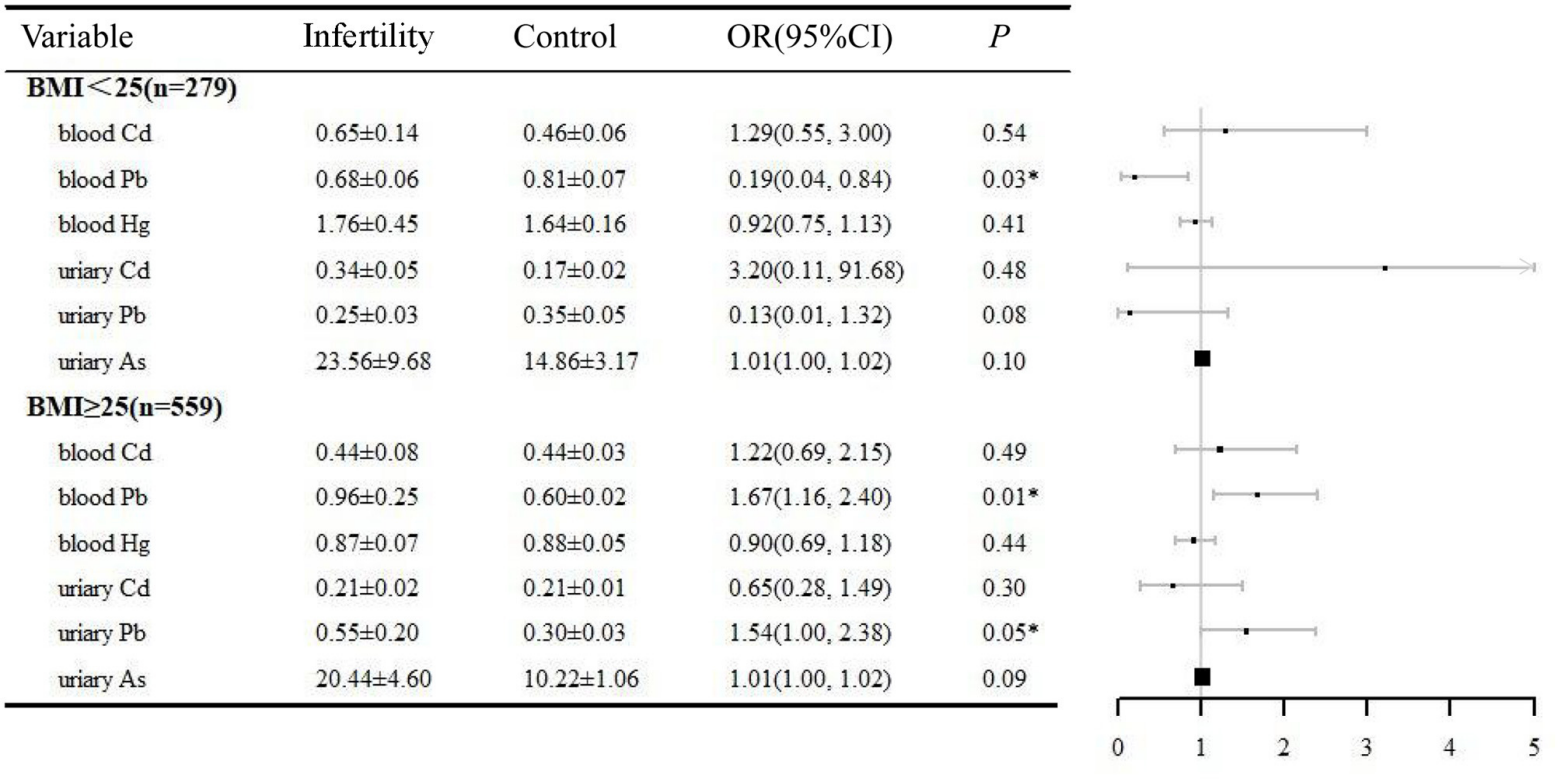
BMI-stratified analysis of the correlation between heavy metals and infertility, NHANES 2013–2018. *P < 0.05. BMI, body mass index; OR, odds ratio; CI, confidence interval.

Both age and BMI are important factors contributing to female infertility. In this study, a stratified analysis was performed based on the above two factors. The relationship between heavy metals exposure and infertility was analyzed in logistic regressions adjusted by BMI (or age), ethnicity, education, marital status, PIR, BMI, regular menstrual periods, pelvic infection, and smoking history (Figures 2, 3). In the women aged 35–44 years, urinary Pb (OR = 1.52, 95% CI 1.07, 2.16), blood Pb (OR = 1.68, 95% CI 1.11, 2.25), and urinary As (OR = 1.02, 95% CI 1.00, 1.03) were positively correlated with infertility. However, heavy metal exposure was not associated with infertility in women aged 20–34 years. Blood Pb (OR = 1.67, 95% CI 1.16, 2.40) and urine Pb (OR = 1.54, 95% CI 1.00, 2.38) were positively related with infertility in women with BMI ≥25. It is noteworthy that blood Pb (OR = 0.19, 95% CI 0.04, 0.84) was negatively correlated with infertility in women with BMI < 25, which need to be further validated.

## Discussion

This study extracted 838 samples from NHANES 2013–2014, 2015–2016, and 2017–2018 cycles to evaluate the correlation between heavy metal exposure and female infertility. Urinary As was significantly associated with female infertility, and the risk of infertility increased with higher urinary As levels. To some extent, urinary Cd was correlated with infertility. Blood/urine Pb was related with infertility in advanced age and overweight/obese women.

In clinical practice, human blood and urine are usually tested to evaluate heavy metal levels. Blood tests respond to the retention of heavy metals in the circulatory system. However, heavy metals only circulate in the blood for a short period before entering the tissues. The urine test refers to a response to the concentration of heavy metals stored in the body's urine at a particular moment. It can reflect the high load of heavy metals exposed to the body. In this study, As and Cd in urine were positively correlated with female infertility, whereas there was no correlation in blood. Both blood and urinary Pb were positively related with advanced and overweight/obese female infertility. These findings suggested that comparing the results among different samples is more reliable than assessing human heavy metal exposure.

As, a by-product of industrial processes, has been confirmed as a common contaminant in soil and groundwater. High levels of As have been detected in poultry fed with drugs containing As and in rice in some areas. Moreover, As can produce reactive oxygen species. Oxidative stress results from the oxidation of arsenite to arsenate *via* As methylation. It impairs the physiological functions of cells and induces various diseases (e.g., cancer, diabetes, atherosclerosis, cardiovascular disease, and infertility). As was also an endocrine disruptor for causing sex hormone changes. Decreased serum estradiol (E2), luteinizing hormone (LH) and follicle-stimulating hormone (FSH) levels, reduced ovarian and uterine weights, and fewer healthy follicles contributed to the development of follicular atresia, reduced endometrial glands and thinning of the myometrium in female rats exposed to drinking water (4 μg/mL or 0.4 ppm), which were correlated with the down-regulation of estrogen receptors and downstream responsive genes (29, 30). Three-week-old SD rats were exposed to As-containing drinking water (0, 0.02, 0.2, and 2 mg/L) for 44 days, and the results of the study were approximately the same as before. However, arsenic increased LH and FSH levels in a dose-dependent manner, and the mechanism of action may be related to the selective downregulation of ovarian steroid-related proteins (31). Relevant epidemiological investigations from Taiwan, China have suggested that blood concentrations of As were significantly higher in infertile women than in pregnant women (14). Pinchoff et al. (32) examined reproductive outcomes in Indian women and found that As-exposed areas exhibited higher rates of stillbirth, recurrent miscarriage, and infertility compared with unexposed areas. A study reported no correlation between blood As and fertility in New York State women (33). Another study has suggested that there was no significant difference in endometrium As concentration between normal women and women with unexplained infertility (34). This study was the first to report a significant positive correlation between urinary As and female infertility, correlated with advanced age factors.

Pb, one of the three major heavy metal pollutants, refers to a heavy metal element that seriously endangers human health. The ideal amount of Pb in the human body is zero. Humans mostly bring lead into the body by ingesting food and drinking tap water. Serotonin (5-HT) and norepinephrine (NE) were reduced, whereas LH and FSH did not vary after 0.05 mg/kg Pb intervention in estrous rats (35). The possible reason for decreased E2 concentrations and reduced numbers of mature oocytes found in Taranto women is the long-term exposure to Pb (36). Blood Pb levels showed statistically significant and negative correlations with MII oocyte count, implantation, clinical pregnancy, and sustained pregnancy rates in patients subjected to unexplained infertility (37). An epidemiological survey from Taiwan, China has shown that blood Pb concentrations were significantly higher in infertile women than in pregnant women (14). Guerra-Tamayo et al. (38) found that exposure to high Pb concentrations may be an essential risk factor affecting the period of pregnancy in women, especially in fertile women who have been trying to conceive for more than 1 year, through a follow-up study over 4 years. In this study, although blood and urinary Pb levels were not associated with infertility, they were significantly and positively correlated with infertility in older and overweight/obese women. Findings of this study were similar to the literature 38 and 40, which suggest that older and overweight/obese infertile women should be aware of Pb exposure.

Cd is not a critical element in the human body. It is absorbed from the external environment after birth, largely through food, water, and air, into the body to accumulate down. In male infertility, Cd is classified as a highly toxic element. Chabchoub et al. (39) examined the levels of Pb, Cd, zinc, and copper in the blood and urine of subjects through atomic absorption spectrophotometry. They have noted that urinary cadmium levels were positively correlated with abnormal sperm morphology. 5-HT, NE, dopamine, LH, and FSH were reduced after 0.05 mg/kg Pb intervention in estrous rats (35). Lei et al. (14) found significantly higher blood concentrations of Pb and As, rather than Cd, in infertile women than in pregnant women. In this study, blood and urinary Cd were not correlated with infertility after adjusting for all covariates factors, but urinary Cd was positively correlated with female infertility in the crude model, model 1, and model 2. In the present study, blood and urinary Cd were not correlated with infertility after adjusting for all covariates factors, which was similar to the literature 28. However, Cd in urine was positively associated with female infertility in the crude model, model 1 and model 2.

This study also has some limitations. First, since this study followed a cross-sectional design, it revealed that Cd, Pb, and As were correlated with female infertility, whereas the causal relationship was not explained. Second, the infertility judgments originated from a self-reported questionnaire. Although self-reported infertility is a valid method of determination, the presence of infertility in the male partner and memory confusion of the time of preparation may affect the diagnosis of female infertility, thus causing biased results. Third, other confounding variables (e.g., birth history, frequency of intercourse, and contraceptive use) were not covered. Lastly, the results would have been of great practical significance if a graph or table depicting the geographical distribution of the final sample had been included. In brief, the results of this study should be validated in depth through subsequent prospective studies.

## Data availability statement

Publicly available datasets were analyzed in this study. This data can be found here: https://www.cdc.gov/nchs/nhanes/index.htm.

## Ethics statement

The studies involving human participants were reviewed and approved by the National Center for Health Statistics Research Ethics Review Board. Written informed consent for participation was not required for this study in accordance with the national legislation and the institutional requirements.

## Author contributions

JL and JX contributed to conception and design of the study. JL and XL organized the database and wrote the first draft of the manuscript. XL performed the statistical analysis. JL, XL, JQ, and XY wrote sections of the manuscript. All authors contributed to the article and approved the submitted version.
